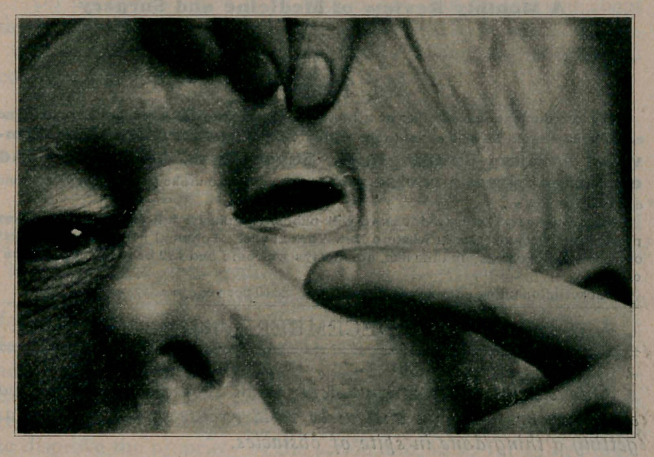# Primary Melanotic Sarcoma of Iris with Metastasis to Liver

**Published:** 1912-12

**Authors:** 


Primary Melanotic Sarcoma of Iris with Metastasis to Liver, Geo. F. Suker and L. N. Grosvenor of Chicago, *Ill. Med. Jour.*, Oct., 1912. Female, small black spot in iris first noticed in 1901 at age of 23, operation followed by X-ray treatment for 18 months. Recurrence and operation in 1904. Recurrence and nostrum application in 1905. (Note by editor: However foolish, can we blame the patient?) In 1906 "therapeutic light" treatments. July 12, 1907, Suker did a complete exenteration of the orbid and sinuses. December, 1907, on account of abdominal symptoms, exploratory laparotomy by Grosvenor, radical operation impossible. Death April 23, 1908. Through the courtesy of the authors and the editor, the illustrations are reproduced.

## Figures and Tables

**Figure f1:**
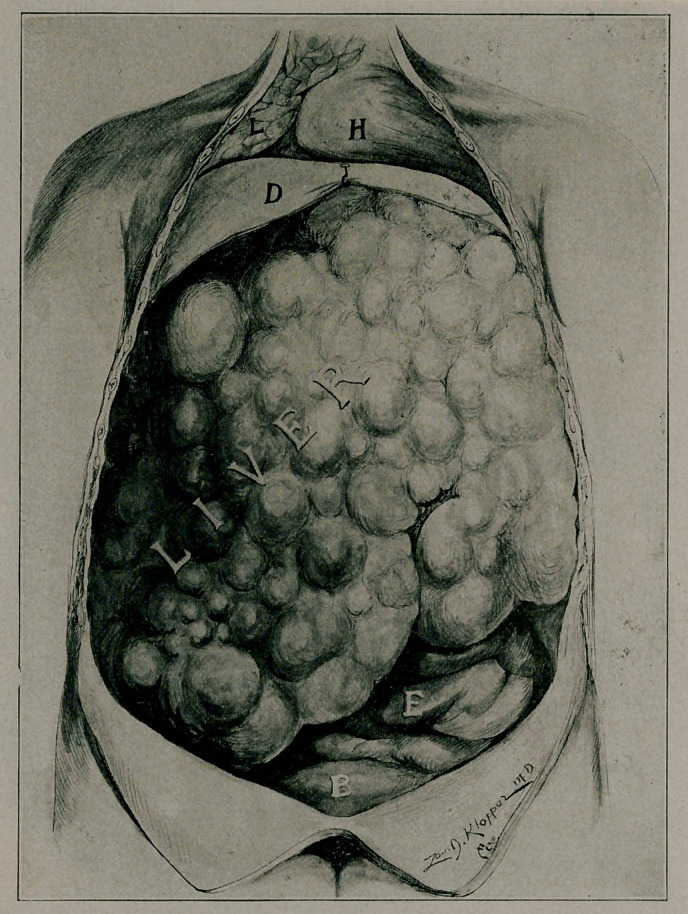


**Figure f2:**